# Novel Insights into Amlodipine-Induced Gingival Enlargement: A Clinical and Molecular Perspective

**DOI:** 10.3390/ph17081075

**Published:** 2024-08-16

**Authors:** Jana Mojsilović, Nemanja Jovičić, Sanja Vujović Ristić, Momir Stevanović, Sara Mijailović, Gvozden Rosić, Slobodan Janković, Marina Kostić

**Affiliations:** 1 Department of Dentistry, Faculty of Medical Sciences, University of Kragujevac, Svetozara Markovića 69, 34000 Kragujevac, Serbia; sanja.994@live.com (S.V.R.); momirstevanovic7@gmail.com (M.S.); 2 Department of Histology and Emrbiology, Faculty of Medical Sciences, University of Kragujevac, Svetozara Markovića 69, 34000 Kragujevac, Serbia; 3 Department of Medical Statistics and Informatics, Faculty of Medical Sciences, University of Kragujevac, Svetozara Markovića 69, 34000 Kragujevac, Serbia; saramijailovic212@gmail.com; 4 Department of Physiology, Faculty of Medical Sciences, University of Kragujevac, Svetozara Markovića 69, 34000 Kragujevac, Serbia; grosic@medf.kg.ac.rs; 5 Department of Pharmacology and Toxicology, Faculty of Medical Sciences, University of Kragujevac, Svetozara Markovića 69, 34000 Kragujevac, Serbia; slobnera@gmail.com (S.J.); marrina2006kg@yahoo.com (M.K.); 6 Center for Harm Reduction of Biological and Chemical Hazards, Faculty of Medical Sciences, University of Kragujevac, Svetozara Markovića 69, 34000 Kragujevac, Serbia

**Keywords:** amlodipine, gingival overgrowth, oxidative stress, quality of life

## Abstract

This study aimed to identify risk factors for amlodipine-induced gingival enlargement, assess quality of life, and analyze gingival tissue. This cross-sectional study involved hypertensive patients on amlodipine, divided into groups with and without gingival enlargement. Assessments included sociodemographic data, clinical evaluations, and clinical parameters. Quality of life was assessed using OHIP-14 and WB-HRQoL scales. Gingival tissue samples were analyzed for oxidative status and key molecules using RT-PCR and colorimetric assays. The study included 32 patients with no significant sociodemographic differences between groups (*p* > 0.05). Patients with gingival enlargement had higher systolic blood pressure (139.63 ± 10.743 vs. 128.38 ± 7.249, *p* = 0.028) and higher OHIP-14 scores. The RT-PCR analysis showed significant differences in IL-6, TNF-α, IL-33, ST2, TGF-β1, FGF-2, CTGF, VEGF-D, and KGF expression. IL-6, TNF-α, ST2, and FGF-2 expression levels were lower in patients taking amlodipine, with and without gingival enlargement. TGF-β1 and CTGF expression levels were highest in patients with amlodipine-induced gingival enlargement. SOD activity was also highest in these patients, whereas MDA levels were higher in patients with gingival enlargement without amlodipine. Our study highlights the impact of amlodipine-induced gingival enlargement on oral health and quality of life, emphasizing fibrosis and oxidative stress, and suggests the need for integrated healthcare approaches and further research.

## 1. Introduction

Drug-induced gingival enlargement is a recognized side effect associated with various medications, including anticonvulsants, immunosuppressants, and calcium channel blockers [1]. Calcium channel blockers, a class frequently employed in the management of hypertension (37%), chronic, stable, and vasospastic angina, and cardiac arrhythmias, exert their therapeutic effects by blocking voltage-gated calcium channels. This action results in diminished arterial contractions, reduced arterial pressure, and enhanced myocardial perfusion [2]. However, the utilization of calcium channel blockers is often constrained by the prevalence of side effects, with the most common manifestations being headache, swelling, constipation, palpitations, nausea, dyspepsia, and gingival enlargement [2,3,4]. Notably, nifedipine is associated with gingival enlargement in 43.6% of cases, while amlodipine induces it in 26.7% of cases. The recent literature indicates an upward trend for amlodipine-induced gingival enlargement, with frequencies reaching as high as 61.8% [3,4,5]. Amlodipine, belonging to the third generation of this drug class, is relatively novel and exhibits fewer severe side effects in comparison to its predecessors [2,6,7,8,9]. The gingival enlargement presents a substantial functional and aesthetic concern, impeding proper hygiene maintenance and potentially leading to tooth loss, which can significantly compromise the patient’s quality of life. This adverse effect can present with increased clinical bleeding, and management strategies involving products like hyaluronic acid or lactoferrin might offer benefits in controlling gingival inflammation and bleeding, as demonstrated in recent randomized controlled trials [10]. Moreover, the recurrence of gingival enlargement after surgical intervention is commonplace, further diminishing the overall efficacy of calcium channel blockers in hypertension treatment and adversely affecting the quality of life for affected patients [6]. It is crucial to consider these implications when assessing the risk–benefit profile of calcium channel blockers in clinical practice.

Potential mechanisms for amlodipine-induced gingival enlargement can be categorized into inflammatory and non-inflammatory pathways. Non-inflammatory mechanisms include the following: (1) a decrease in sodium flux by the drug, leading to reduced cellular folate uptake and subsequent collagenase deficiency, resulting in impaired connective tissue catabolism and the clinical presentation of drug-induced gingival enlargement; (2) an increase in adrenocorticotropic hormone levels due to the blocking of synthesis in the adrenal cortex; and (3) the upregulation of transforming growth factor-beta 1 (TGF- β1) due to inflammation in the gingival cervical fluid. Inflammatory mechanisms involve the following: (1) the presence of concentrated drug in the cervical gingival fluid, resulting in localized inflammatory effects; and (2) upregulation of keratinocyte growth factor (KGF) [11]. Various factors, including age, gender, medications, genetic predisposition, and periodontal health, have been linked to the development of gingival enlargement [12]. Most research focused on the cells and extracellular components of gingival tissues, emphasizing the critical role of cytokines and growth factors in extracellular matrix synthesis. These molecules also influence cell proliferation and differentiation within the connective tissue, and changes in cytokine profiles and inflammatory cell populations have been identified in previous studies [6].

While the risk factors for drug-induced gingival enlargement are well-established, the precise mechanistic underpinnings of this side effect remain elusive. Previous in vitro studies have underscored the pivotal role of inflammatory cytokines, particularly interleukin-1 (IL-1) and interleukin-6 (IL-6), in the connective tissue in response to cyclosporine exposure [6]. Furthermore, these studies have revealed a reduction in matrix metalloproteinases 1 and 3 in fibroblasts within the enlarged gingiva due to cyclosporine therapy [6]. Notably, cyclosporine has been demonstrated to induce the generation of intracellular reactive oxygen species (ROS) and fortify the extracellular matrix’s structural integrity, thereby enhancing resistance to protease activity [13,14,15]. The investigation by Sam Becerik et al. elucidates that cyclosporine administration leads to an elevation in transglutaminase 2 (TGM-2) levels in gingival cervical fluid and plasma. TGM-2, in turn, influences cyclosporine-induced gingival enlargement by modulating the oxidative status in gingival cervical fluid and plasma [15]. Yu-Tang Chin and colleagues’ study [10] involving primary fibroblast cell culture and laboratory animals indicates that cyclosporine stimulates ROS production, with clinical improvement observed upon antioxidant (vitamin E and sulforaphane) use, highlighting the role of oxidative stress in the pathogenesis of cyclosporine-induced gingival enlargement. A pilot study on 10 subjects suggests a significant involvement of TGF-β1 in the pathogenesis of amlodipine-induced gingival enlargement [3]. Siddika Selva Sume et al.’s recent publication establishes that independent of inflammation, amlodipine elevates interleukin-17A concentration in gingival tissue, potentially instigating fibrotic changes in the gingival tissue [16]. Overall, these previous studies propose that these alterations in cell populations and cytokine levels affect gingival tissue response and may play a role in the mechanism of gingival enlargement.

However, comprehensive investigations into the association between risk factors and the clinical presentation, quality of life, oxidative stress parameters, and relative gene expression for relevant molecules in gingival enlargement in patients under chronic amlodipine administration remain insufficiently explored. Further research in this population is warranted to enhance our understanding of these complexities. The objective of this investigation was to identify risk factors contributing to the development of gingival enlargement in response to the chronic administration of amlodipine. Additionally, the study aimed to assess the quality of life, analyze tissue oxidative stress parameters, and explore relative gene expression for relevant molecules in tissues from patients undergoing chronic amlodipine therapy.

## 2. Results

### 2.1. Sociodemographic Characteristics, Clinical Characteristics, and Quality of Life

The study included 32 patients, with 11 males and 21 females. No statistically significant difference was observed in any of the examined sociodemographic characteristics between the two groups, one with gingival enlargement and the other without gingival enlargement (*p* > 0.05) (Table 1).

Patients with and without gingival enlargement did not differ in terms of medication use and dosage, duration of medication use in months, disease duration in years, or diastolic blood pressure. However, patients with gingival enlargement had a significantly higher systolic blood pressure (139.63 ± 10.743) compared to patients without gingival enlargement (128.38 ± 7.249) (*p* = 0.028) (Table 2).

Patients with and without gingival enlargement differed in their scores on the OHIP-14 scale and PBI values (Table 3).

Patients with and without gingival enlargement differed in terms of masticatory difficulties (Table 4).

The patients (n = 24) were further categorized into three distinct groups: (1) patients taking amlodipine who developed gingival enlargement (A+GE+), (2) patients not taking amlodipine or any other medication but who developed gingival enlargement (A−GE+), and (3) patients taking amlodipine who did not develop gingival enlargement (A+GE−). This categorization was implemented to specifically focus on the investigation of gingival tissue, as the inclusion of patients without gingival enlargement was deemed irrelevant to this analysis.

### 2.2. Cytokine Profile in Gingival Tissue

**Figure 1:**
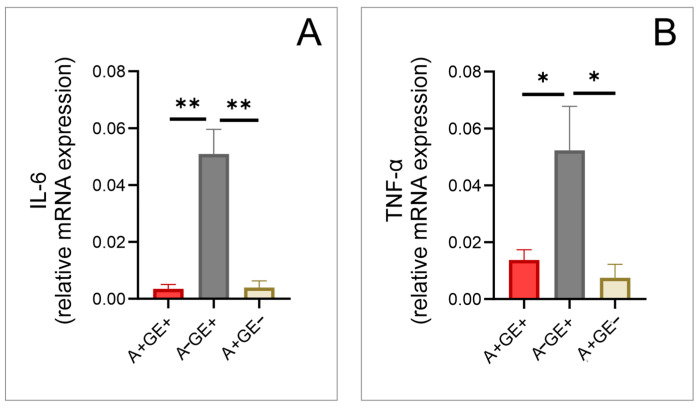
Inflammation in gingival tissue (relative gene expression; three equal groups, n = 24; A+GE+—amlodipine with gingival enlargement; A−GE+—gingival enlargement without amlodipine; A+GE−—amlodipine without gingival enlargement). (**A**) IL-6; (**B**) TNF-α. The values are presented as mean ± SD (* *p* < 0.05, ** *p* < 0.01).

In the gingival tissue samples, the RT-PCR analysis demonstrated a significant difference in the expression levels of IL-6 and tumor necrosis factor-α (TNF-α). Specifically, IL-6 and TNF-α expression levels were lower in patients taking amlodipine compared to those with gingival enlargement who were not taking amlodipine (Figure 1).

**Figure 2:**
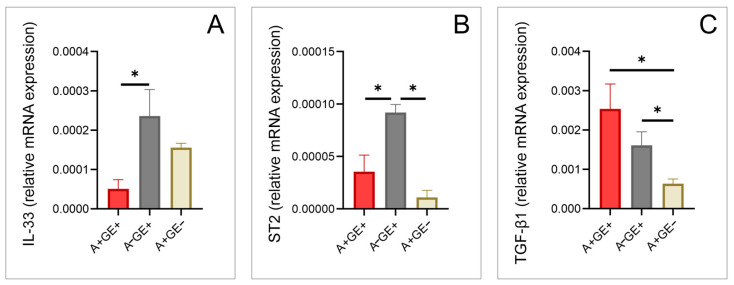
Cytokine alterations in gingival tissue (relative gene expression; three equal groups, n = 24; A+GE+—amlodipine with gingival enlargement; A−GE+—gingival enlargement without amlodipine; A+GE−—amlodipine without gingival enlargement). (**A**) IL-33; (**B**) ST2; and (**C**) TGF-β1. The values are presented as mean ± SD (* *p* < 0.05).

The RT-PCR analysis of gingival tissue samples showed that interleukin-33 (IL-33) expression levels were significantly lower in patients taking amlodipine who developed gingival enlargement compared to those not taking amlodipine but who developed gingival enlargement (Figure 2).

The suppression of tumorigenicity 2 receptor (ST2) expression levels were found to be significantly different between the group of patients taking amlodipine who developed gingival enlargement compared to the patients not taking amlodipine but had gingival enlargement, as well as between the patients not taking amlodipine that had gingival enlargement and the patients taking amlodipine without this adverse effect (Figure 2).

Significant differences in the expression of TGF-β1 were observed, as depicted in Figure 2. Patients taking amlodipine who developed gingival enlargement had significantly higher TGF-β1 expression levels than those not taking amlodipine but with gingival enlargement, as well as those taking amlodipine without gingival enlargement. Additionally, patients not taking amlodipine who had gingival enlargement showed significantly higher TGF-β1 expression levels than those taking amlodipine without gingival enlargement.

### 2.3. Relative Expression of Growth Factors in Gingival Tissue

**Figure 3:**
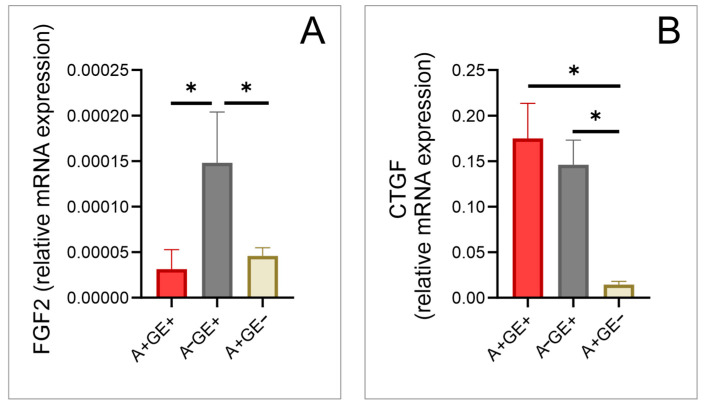
Alteration of Growth Factors in Gingival Tissue (relative gene expression; three equal groups, n = 24; A+GE+—amlodipine with gingival enlargement; A−GE+—gingival enlargement without amlodipine; A+GE−—amlodipine without gingival enlargement). (**A**) FGF2; (**B**) CTGF. The values are presented as mean ± SD (* *p* < 0.05).

Marked differences in the expression of fibroblast growth factor 2 (FGF-2) were found between the group of patients taking amlodipine who developed gingival enlargement compared to those not taking amlodipine but had gingival enlargement, as well as between the patients not taking amlodipine that had gingival enlargement and the patients taking amlodipine without gingival enlargement (Figure 3).

There were significant differences in the expressed levels of connective tissue growth factor (CTGF) between the group of patients taking amlodipine who developed gingival enlargement compared to the patients taking amlodipine without gingival enlargement, as well as between the patients not taking amlodipine that had gingival enlargement and the patients taking amlodipine without gingival enlargement (Figure 3).

**Figure 4:**
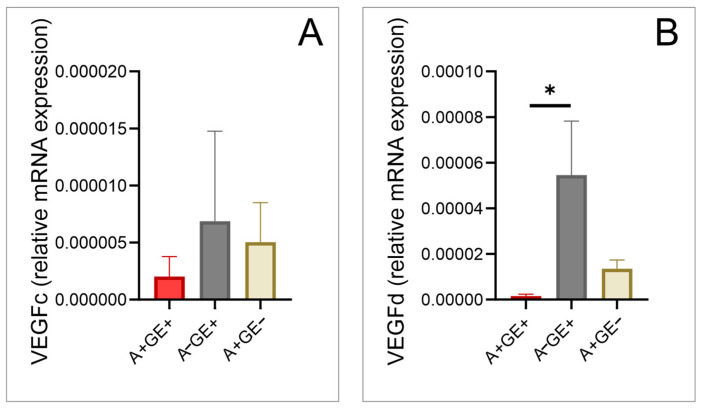
Alteration of growth factors in gingival tissue (relative gene expression; three equal groups, n = 24; A+GE+—amlodipine with gingival enlargement; A−GE+—gingival enlargement without amlodipine; A+GE−—amlodipine without gingival enlargement). (**A**) VEGF-C; (**B**) VEGF-D. The values are presented as mean ± SD (* *p* < 0.05).

**Figure 5:**
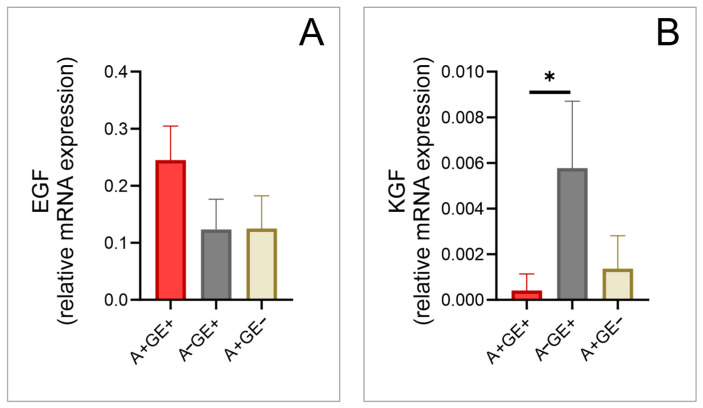
Alteration of growth factors in gingival tissue (relative gene expression; three equal groups, n = 24; A+GE+—amlodipine with gingival enlargement; A−GE+—gingival enlargement without amlodipine; A+GE−—amlodipine without gingival enlargement). (**A**) EGF; (**B**) KGF. The values are presented as mean ± SD (* *p* < 0.05).

No significant differences were observed in the expression of vascular endothelial growth factor C (VEGF-C) (Figure 4) and epidermal growth factor (EGF) among the studied groups (Figure 5).

Both vascular endothelial growth factor D (VEGF-D) (Figure 4) and KGF showed significant differences between patients taking amlodipine with gingival enlargement and those not taking amlodipine (Figure 5).

### 2.4. Oxidative Status

**Figure 6:**
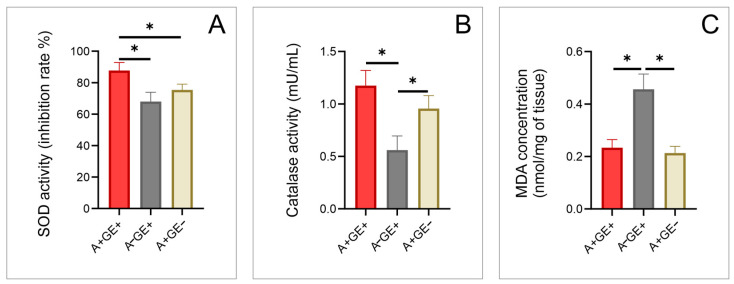
Oxidative status markers (three equal groups, n = 24; A+GE+—amlodipine with gingival enlargement; A−GE+—gingival enlargement without amlodipine; A+GE−—amlodipine without gingival enlargement). (**A**) SOD activity; (**B**) Catalase activity; and (**C**) MDA concentration. The values are presented as mean ± SD (* *p* < 0.05).

Using a colorimetric assay kit, superoxide dismutase (SOD) levels showed a statistically significant difference between the groups of patients taking amlodipine who developed gingival enlargement compared to those taking amlodipine without gingival enlargement (Figure 6).

Catalase (CAT) levels and malondialdehyde (MDA) levels were both found to be statistically different between the group of patients taking amlodipine who developed gingival enlargement compared to the patients not taking amlodipine but who had gingival enlargement, as well as between the patients not taking amlodipine that had gingival enlargement and the patients taking amlodipine without gingival enlargement (Figure 6).

## 3. Discussion

The adverse effects of drugs on oral health are substantial not only in terms of clinical manifestations but also regarding their multifaceted impact on patients’ daily lives by interfering with numerous physiological functions. Despite the diverse clinical presentations of adverse drug reactions affecting oral health, available medical documentation lacks a dedicated section addressing drug effects on the oral mucosa. Consequently, the role of dentists in the timely detection of these side effects within modern healthcare systems is crucial. Furthermore, the professional community’s needs for assessing adverse drug reactions on oral health extend beyond the presentation of clinical manifestations and require a comprehensive evaluation of these phenomena [17,18,19].

Gingival enlargement can be caused by the use of over 30 different groups of drugs, among which amlodipine, a calcium channel blocker, occupies a prominent position as an etiological agent due to its widespread use in the management of hypertension, a highly prevalent condition. The goals of this research were to evaluate the key factors contributing to gingival enlargement in patients on chronic amlodipine therapy and to assess both the local and systemic consequences of this side effect.

The results of our study revealed a statistically significant difference in systolic pressure values in patients with gingival enlargement. Although patients with gingival enlargement initially achieved target hypertension values (below 140/90 mmHg and 140/85 mmHg for those with cardiovascular or chronic kidney disease and diabetes mellitus, respectively), the discrepancy can be explained by other group characteristics. Specifically, patients with gingival enlargement had a longer duration of hypertension and were treated with amlodipine, either alone or in combination, for a more extended period, likely due to uncontrolled hypertension. Moreover, the presence of adverse oral effects, such as gingival enlargement, has been shown to increase the risks of cardiovascular diseases. This connection underscores the interrelationship between oral health and systemic health. Poor oral health and oral adverse effects can exacerbate systemic conditions, creating a bidirectional link where the management of one condition directly impacts the other. This highlights the need for integrated healthcare approaches that address both oral and cardiovascular health concurrently [20,21].

Our results did not show a correlation between dose regimen, duration of disease and therapy, and the occurrence of amlodipine-induced gingival enlargement, which is consistent with the findings of Umeizudike KA et al. [22] and Guttal KS et al. [18].

Our findings revealed statistically significant increases in OHIP-14 scores among patients with gingival enlargement, indicating a decreased quality of life in this population. These results can be attributed to the fact that gingival enlargement, as a manifestation of the adverse effects of calcium channel blockers, has both clinical and functional significance. It interferes with occlusion, nutrition, and oral hygiene, and can lead to tooth loss. Additionally, the presence of gingival enlargement contributes to the development of severe forms of periodontal disease, which is itself a risk factor for numerous cardiovascular diseases [22]. Besides impairing function and increasing the risk of cardiovascular disease, gingival enlargement also poses a notable aesthetic concern for patients; therefore, this local adverse effect of amlodipine should be considered significant in terms of its impact on general health [23,24].

Initially, our study categorized patients into two groups based on gingival enlargement presence to explore medication-related differences. The subsequent analysis revealed distinct tissue characteristics with amlodipine therapy, refining our cohorts into three groups (n = 24) for detailed examination. This focused approach allowed us to investigate specific molecular features associated with amlodipine. Patients without gingival enlargement and not using amlodipine (n = 8) were excluded from detailed analysis as their inclusion did not align with the study’s primary objectives. This study aimed to uncover the mechanisms underlying this adverse effect, particularly in chronic amlodipine users, by analyzing gingival tissue samples to identify key factors contributing to gingival enlargement and explore how amlodipine may influence them.

In order to investigate the impact of the inflammatory component in the pathogenesis of amlodipine-induced gingival enlargement, we examined the relative gene expression of IL-6 and TNF-α. These cytokines were selected for examination due to their known involvement in inflammatory processes and modulation by amlodipine, as highlighted in previous studies [6,25,26]. Our results showed that the expression of these cytokines was reduced in patients taking amlodipine, regardless of whether they developed this side effect or not. Our results align with previous studies that examined the expression and the levels of these cytokines in blood plasma before and after amlodipine administration in patients suffering from atherosclerosis and congestive heart failure [25,26]. The reduced gene expression of these two key inflammation mediators, compared to patients with gingival enlargement caused by inflammatory reactions rather than drug use, suggests that amlodipine may have a partially anti-inflammatory effect [25] within gingival tissue as well. Furthermore, this finding indicates that inflammation may not play a crucial role in the onset of this side effect, even though it could exacerbate and lead to more severe consequences in the more progressive stages of the condition, as the previous studies concluded [2]. This is particularly relevant since amlodipine-induced gingival enlargement can impair oral hygiene, leading to inflammation on top of the existing condition [2]. Importantly, among patients not receiving amlodipine but showing enlargement, where these cytokine expression levels were most pronounced, there was also the highest PBI score, a sensitive indicator of gingival inflammation severity, indicating consistency between the clinical presentation and tissue analysis.

Given IL-33’s unexplored role in amlodipine-induced gingival enlargement and its known involvement in fibrosis and inflammatory processes [27,28,29,30], it was important to examine its expression in these cases. The lower cytokine expression levels observed in patients using amlodipine, particularly those with gingival enlargement, may be explained by previous findings showing increased IL-33 expression during the advanced or stable phase of periodontal disease when a Th2 inflammatory response dominates, probably due to IL-33’s profibrotic role [27,28,29,30]. However, our tissue analysis did not find evidence of this during the phase we studied; instead, elevated expression levels of TGF-β1 indicate its likely involvement in the fibrotic component of the pathogenesis of this side effect. Similar to previous studies focused on gingival cervical fluid analysis, our analysis of gingival tissue revealed the increased expression of TGF-β1 and CTGF in patients with gingival enlargement who were taking amlodipine. Our results support the theory that TGF-β1 may lead to the accumulation of the extracellular matrix by stimulating fibroblasts and inhibiting matrix degradation, thereby resulting in gingival enlargement [3,31,32]. Also, the collective activity of these growth factors may potentially play a pivotal role in the onset of amlodipine-induced gingival enlargement, considering the presence of the fibrotic component inherent in this adverse effect. Our results also prompt consideration of whether amlodipine itself influences the increased production of TGF-β1, as demonstrated in previous studies involving mononuclear cells [33]. However, since the expression of TGF-β1 is elevated in the group of patients with gingival enlargement without amlodipine therapy and significantly lower in the group without enlargement but taking the medication, further research on the direct impact of the drug on these growth factor levels would be necessary.

We selected the relative gene expressions of FGF-2 and KGF for examination due to their recognized importance in tissue repair mechanisms and their interactions with gingival epithelial cells and fibroblasts, as documented in previous research [34,35,36] that may be relevant for gingival enlargement. Our research demonstrated significantly lower expressions of fibroblast growth factors in patients taking amlodipine, whether or not they developed the side effect, compared to patients with inflammation-induced gingival enlargement. Our findings can be explained by the fact that these growth factors play a significant role in tissue repair following injury or trauma [34], which is certainly the case in gingival enlargement caused by inflammation in patients not taking amlodipine. Additionally, our results align with previous studies suggesting that these two growth factors work in concert with inflammatory cytokines [35,36], as evidenced in our study.

Additionally, VEGF-D exhibits elevated expression levels during inflammatory conditions [37], and we examined it due to its established role in promoting lymphangiogenesis and its association with inflammatory responses [37,38,39], which could provide insights into the mechanisms underlying gingival enlargement. It was significantly higher in patients with inflammation-induced gingival enlargement compared to those with amlodipine-induced gingival enlargement. This suggests that the inflammatory component potentially may not significantly contribute to the onset of amlodipine-induced gingival enlargement. Our results do not indicate an elevated expression level of this growth factor at the local level in individuals taking amlodipine, which diverges from previous study findings [38,39], likely due to variations in tissue types examined, suggesting a complex mechanism of action for this growth factor that future research may elucidate.

Given the well-documented role of oxidative stress in diseases like AIDS, diabetes, cancer, periodontal disease, including gingival inflammation, and fibrotic diseases [15,40], it was crucial to explore its potential involvement in amlodipine-induced gingival enlargement, particularly in the absence of existing data linking these concepts. Previous studies [13,14,15] have found a connection between elevated oxidative stress levels and cyclosporine-induced gingival enlargement, which has a predominantly inflammatory origin due to the drug’s mechanism of action. Other studies [41,42] also suggest that amlodipine itself may have potential antioxidant effects, making it essential to examine these parameters in the gingival tissue of patients taking this medication.

Our investigation revealed a statistically significant difference in SOD activity between patients taking amlodipine with gingival enlargement and those not taking the drug, as well as between patients without gingival enlargement but taking amlodipine. This indicates that the increased activity, or need for the activity of this enzyme, suggests a heightened requirement for oxidative protection due to the increased production of ROS in patients taking amlodipine who developed gingival enlargement, as the enzyme is more active in response to increased superoxide radical production. Given the significantly lower activity of this enzyme in patients without gingival enlargement who are taking the drug, we can infer that one of the reasons for the occurrence of this side effect in only a specific group of patients may potentially be related to increased ROS production. In patients without gingival enlargement, the lower SOD activity reflects a lower oxidative stress level, as there is less need for an enhanced antioxidative response.

Additionally, MDA levels, a marker of oxidative damage, were highest in patients with gingival enlargement not taking amlodipine. The gingival enlargement in this group is caused by inflammation and possibly bacterial infection, leading to increased lipid peroxidation. Consequently, the oxidative protection in these patients is the lowest, resulting in the highest oxidative damage. The highest MDA levels in this group indicate severe oxidative damage due to inadequate oxidative protection in the presence of ongoing inflammation and infection. In the group with gingival enlargement taking amlodipine, MDA levels were lower, which suggests that while oxidative damage is still present, it is less severe, potentially due to the antioxidant effects of amlodipine [41] mitigating some oxidative stress. Amlodipine’s potential antioxidative properties, including its ability to reduce ROS production and enhance antioxidant defenses, may contribute to lowering MDA levels despite the presence of gingival enlargement. This possible antioxidative effect is partially linked to amlodipine’s chemical structure and lipophilicity. Due to its high lipophilicity, amlodipine might be able to penetrate cellular membranes and interact with lipid peroxides, potentially helping to prevent lipid peroxidation. The dihydropyridine ring within amlodipine could play a role in this process by possibly donating protons, which might help stabilize free radicals and disrupt the peroxidation process. Additionally, the aromatic ring structure of amlodipine may attract free radicals, which could further contribute to its antioxidative properties [41]. However, the persistence of elevated MDA levels indicates that oxidative damage remains a contributing factor to gingival enlargement in these patients, albeit to a lesser extent than in those with gingival enlargement not taking amlodipine. This suggests that while amlodipine might reduce oxidative stress and lipid peroxidation, it does not completely eliminate oxidative damage associated with gingival enlargement.

In previous studies [14,43], increased ROS production has been shown to lead to the increased activation of TGF-β1. Our results also show the increased gene expression of TGF-β1 in patients taking amlodipine who have gingival enlargement compared to those taking amlodipine without this side effect. We can conclude that these two factors are potentially connected and that their mutual role might underlie the pathogenesis of this adverse effect. This finding suggests a need for further research to better understand the role of oxidative stress and TGF-β1 in the mechanism of amlodipine-induced gingival enlargement. Such research could contribute to the development of new strategies for preventing and treating this side effect, such as new treatment modalities with antioxidant agents, as proposed by the study investigating the effects of cyclosporine, another drug that can also cause gingival enlargement [14].

Future research could also explore the potential benefits of ozonized substances in modulating gingival bleeding and treating symptoms of gingival enlargement. Recent studies [44,45] have shown promising results in using ozone therapy and ozonized gels for periodontal management since ozone is beneficial across various stages of gingival and periodontal diseases due to its antimicrobial properties, ability to neutralize microbial toxins associated with periodontal issues, and its role in promoting tissue healing and regeneration. Exploring these approaches could provide new therapeutic options for gingival enlargement and improve patient outcomes.

Overall, our findings have important implications for clinical practice. The identification of oxidative stress and TGF-β1 as potential contributors to amlodipine-induced gingival enlargement underscores the importance of monitoring and managing these factors in patients undergoing long-term amlodipine therapy. Clinicians should consider integrating regular assessments of oxidative stress markers and growth factor levels into routine care for patients on amlodipine to better anticipate and manage gingival enlargement. Our research highlights the need for a multidisciplinary approach to the management of amlodipine-induced gingival enlargement, involving both systemic and local treatment strategies. By addressing both the underlying causes and the clinical manifestations of this condition, clinicians can better manage the oral health of patients on amlodipine and improve their quality of life.

## 4. Materials and Methods

The study was conducted as a cross-sectional research project, specifically designed to scrutinize factors linked to the incidence of gingival enlargement in the adult population undergoing chronic amlodipine therapy.

### 4.1. Study Population

The study design encompasses the classification of patients into two distinct groups: those presenting with gingival enlargement and those without gingival enlargement.

### 4.2. Sampling

This comprehensive categorization allows for a nuanced exploration of factors associated with gingival enlargement, considering both amlodipine exposure and the presence or absence of gingival enlargement in the studied population. The respondents were selected from a pool of adult patients of both genders diagnosed with hypertension and undergoing amlodipine treatment for a minimum of 6 months, provided they satisfied the inclusion criteria. The investigator meticulously screened each patient to ascertain adherence to the stipulated inclusion and exclusion criteria. Upon confirmation of eligibility, patients were enrolled in the study, with the final number determined through statistical tests gauging the study’s statistical power.

### 4.3. Inclusion and Exclusion Criteria

The inclusion criteria for respondents were as follows: adult individuals of both genders who had undergone amlodipine treatment for at least 6 months, possessed a minimum of 12 teeth (including all anterior teeth), required surgical intervention for gingival enlargement (gingivectomy, gingivoplasty), or, in the absence of gingival enlargement, necessitated surgical intervention (surgical extraction of the (partly-)impacted third molars, surgical lengthening of the clinical crown, complicated tooth extractions, frenectomy, gingival flap procedures) for another oral pathology.

Exclusion criteria were as follows: pregnant and breastfeeding women, patients who had received antibiotic therapy within the preceding 3 months, individuals with unregulated diabetes mellitus and other systemic diseases confirmed by reviewing their medical documentation, active smokers, and individuals undergoing combined therapy with other medications known to induce gingival enlargement as a side effect, including cyclosporine A, phenytoin, or oral contraceptives.

### 4.4. Study Population Size

The study population size, determined using the ANOVA test (fixed effects, omnibus, one way) in the statistical program G power, was established at 7 patients for each group, totaling 28 subjects. This estimation was informed by the similar research of Syahputra A. et al. [46]. It is emphasized that this study was conducted as an academic, non-profit research endeavor, adhering to the principles of good clinical practice, good laboratory practice, and the Declaration of Helsinki.

### 4.5. Procedure

All research participants provided signed voluntary consent with comprehensive information before their inclusion in the study. The study received approvals from the Ethics Committee of the University Clinical Center Kragujevac (decision number: 01-22-390), the Health Center Kragujevac (decision number: 01-334414), and the Faculty of Medical Sciences of the University of Kragujevac (decision number: 01-13571) for its execution.

For patients meeting the inclusion criteria who voluntarily signed informed consent documents, a comprehensive assessment was conducted. This evaluation included administering a general questionnaire to collect sociodemographic and clinical data, such as gender, age, habits, the primary medical condition for amlodipine prescription, duration and dosage of amlodipine therapy, presence of symptoms, smoking habits, mouth breathing, bruxism, dental restorations, and information on comorbidities and concomitant therapies. Patients’ comorbidities were taken into account and measured using the Charlson Comorbidity Index (CCI) [47]. Additionally, participants completed the Oral Health Impact Profile—OHIP-14 [48] and Questionnaire for measuring health-related quality of life (HRQoL) in adults residing in western Balkan states—WB-HRQoL [49] scales to determine their quality of life, with prior approvals obtained for their use in the study.

Furthermore, all participants underwent clinical evaluations conducted by a researcher who assessed the severity of the clinical presentation of gingival enlargement. The researcher performed a dental examination to determine specific parameters, including the Sillnes Löe Plaque Index (PI), Papillae Bleeding Index (PBI), Hyperplasia Index following the method of Angelopoulos and Goaz, modified by Pernu et al., Gingival Hyperplasia Index as per Seymour et al., and the determination of the level of the clinical attachment.

Gingival tissue samples were obtained from all enrolled patients for the comprehensive assessment of oxidative status (SOD, CAT, MDA) and crucial molecules associated with gingival enlargement (IL-6, TNF-α, FGF-2, VEGF-C, VEGF-D, TGF-β1, CTGF, ST2, KGF, EGF, and IL-33). The analysis of the gingival tissue sample involved the utilization of RT-PCR (Real-time Polymerase Chain Reaction) and colorimetric assays.

### 4.6. RT-PCR

Following surgical intervention, gingival tissue samples from the patients were promptly frozen in liquid nitrogen and preserved until the commencement of the tissue homogenization process. Total RNA extraction from the tissue homogenate, obtained through manual homogenization, was conducted using TRIzol reagent (Invitrogen, Waltham, MA, USA), comprising guanidine thiocyanate and phenol solution, as per the manufacturer’s instructions. Eppendorf tubes with a volume of 1.5 mL were employed for the experiment and filled with the obtained homogenate. Post-mechanical homogenization, 100 µL of bromochloropropane (1˗bromo˗3˗chloropropane, BCP) was introduced to the solution. After vortexing and a 5 min incubation at room temperature, the mixture underwent centrifugation at 12,000 rpm for 10 min at +40C, yielding three layers, with RNA in the uppermost layer, followed by DNA, and proteins. The RNA was transferred to new Eppendorf tubes, and 500 µL of isopropyl alcohol was added to precipitate the RNA of interest. Following a 15 min incubation at room temperature, centrifugation recommenced for 8 min at 12,000 rpm at +40S. The separated sediment underwent a double wash with chilled 70% ethyl alcohol (1 mL), and post-washing, the sediment was air-dried for 5 min at room temperature and reconstituted in nuclease-free water. The RNA concentration and purity were determined through the spectrophotometric measurement of absorbance at 260/280 nm, utilizing an Eppendorf^®^ Biophotometer (Eppendorf, Hamburg, Germany).

For the reverse transcription process, iScript Re-verse Transcription Mastermix (Bio-Rad, Hercules, CA, USA) was employed, and the RT-PCR reaction utilized SsoAdvanced Universal SYBR Green Supermix (Bio-Rad, USA) and mRNA-specific primers (Table 5) for crucial molecules, namely IL-6, TNF-α, FGF-2, VEGF-C, VEGF-D, TGF-β1, CTGF, ST2, KGF, EGF, and IL-33. Housekeeping genes, β-actin, and glyceraldehyde 3-phosphate dehydrogenase (GAPDH) were included. Quantitative RT-PCR reactions were conducted in Applied Biosystems 7500 (Applied Biosystems, Waltham, MA, USA), and subsequent to the analysis results, the relative gene expression was quantified following the Livak and Schmittgen (2008) methodology [50].

### 4.7. Colorimetric Assays

The evaluation of superoxide dismutase (SOD) activity in gingival tissue samples was conducted using the superoxide dismutase (SOD) activity assay kit (Colorimetric) (ab65354, Abcam, Cambridge, UK). Tissue processing followed the manufacturer’s guidelines, involving harvesting and homogenizing samples in the ice-cold assay buffer provided in the kit. After centrifugation to remove debris, supernatants containing the target enzyme were analyzed. In the colorimetric assay, supernatant samples were incubated with specific substrates, and absorbance was measured at 440 nm using a microplate reader. SOD activity was determined by comparing absorbance readings to a standard curve generated from known SOD concentrations provided in the kit, with results expressed in units per milligram of protein.

For the assessment of catalase activity in gingival tissue samples, the catalase activity assay kit (Colorimetric/Fluorometric) (ab83464, Abcam, Cambridge, UK) was employed. Tissue processing involved homogenization in the ice-cold assay buffer supplied in the kit, followed by centrifugation to collect supernatants containing catalase. In the colorimetric assay, samples were incubated with provided substrates, and absorbance was measured at 570 nm using a microplate reader. Catalase activity was calculated based on the standard curve provided in the kit, with results expressed as units per milligram of protein.

Lipid peroxidation levels in gingival tissue samples were investigated using the lipid peroxidation (MDA) assay kit (ab118970, Abcam, Cambridge, UK). Tissue samples were prepared according to standard procedures, with homogenization in the lysis solution provided in the kit. After centrifugation to remove debris, supernatants were retained for analysis. In the colorimetric assay, supernatant samples were incubated with specific reagents, and absorbance was measured at 532 nm using a microplate reader. MDA concentration, indicative of lipid peroxidation, was determined by comparing absorbance readings with a standard curve generated from known MDA concentrations provided in the kit.

### 4.8. Statistical Analysis

The data were statistically processed using the IBM SPSS Statistics 22 program. The normality of the distribution of continuous variables was initially assessed through the Shapiro–Wilk test. Continuous variables were presented as mean value ± standard deviation or as median (IQR), depending on data normality, while categorical variables were statistically processed by estimating the percentage representation. Subsequently, the Student’s *t*-test was used to compare two groups with normally distributed data, and the Mann–Whitney U test was used to compare non-normally distributed data. The Chi-Square test was used to evaluate the association between categorical variables, and Cramer’s V indicator was used to show the strength of this association. Fisher’s exact test was applied instead of the Chi-Square test when the expected frequencies were less than 5. A statistically significant difference was considered when the *p*-value was less than 0.05.

## 5. Conclusions

In conclusion, our study underscores the significant impact of amlodipine-induced gingival enlargement on both oral health and overall quality of life. The findings highlight the complex interplay between drug therapy, oxidative stress, inflammatory responses, and tissue remodeling processes within gingival tissues. The observed reductions in IL-6 and TNF-α gene expression suggest a potential anti-inflammatory effect of amlodipine at the local gingival level, challenging the notion that inflammation plays a predominant role in the onset of this adverse effect. Moreover, the elevated expression of TGF-β1 and CTGF in patients with amlodipine-induced gingival enlargement points towards fibrosis and extracellular matrix accumulation as key mechanisms contributing to tissue enlargement. Our findings align with previous research, which suggests that amlodipine-induced gingival enlargement may predominantly involve non-inflammatory pathways, while acknowledging that both inflammatory and non-inflammatory mechanisms may be relevant. These insights into the molecular changes and mechanisms highlight the complexity of amlodipine’s effects and underscore the need for further research to explore these pathways in more detail.

Our results also reveal clinically relevant insights, such as the association between systolic pressure levels and the development of gingival enlargement, emphasizing the importance of blood pressure control in managing patients on chronic amlodipine therapy. Furthermore, the significant increase in OHIP-14 scores among patients with gingival enlargement underscores the adverse impact of this condition on quality of life, affecting oral hygiene, nutrition, and aesthetic concerns. These clinical characteristics highlight the multifaceted nature of drug-induced gingival enlargement and its implications for both oral health and systemic well-being. Additionally, our study implicates oxidative stress, indicated by heightened SOD activity in patients with gingival enlargement, suggesting increased ROS production in this cohort. This oxidative stress may further exacerbate tissue damage and contribute to the pathogenesis of gingival enlargement. Importantly, these findings underscore the need for integrated healthcare approaches that address both oral health and systemic implications, particularly in managing patients on chronic amlodipine therapy. Moving forward, further research is warranted to elucidate the precise mechanisms linking amlodipine therapy, oxidative stress, and fibrotic responses in gingival tissues. Future studies could explore novel therapeutic strategies targeting oxidative stress pathways to mitigate adverse oral effects associated with amlodipine use. This type of research can enhance awareness of adverse oral effects among dental professionals and encourage a more proactive role in the system of pharmacovigilance. Such endeavors hold promise for enhancing patient care and minimizing the impact of drug-induced gingival enlargement on oral health and overall well-being.

### Limitations

Our study is constrained by the sample size, which may limit the broader applicability and statistical robustness of our findings. The sample was not randomized due to the limited availability of patients, which introduces a potential selection bias. Additionally, being a unicentric study, local treatment practices may have influenced the results, affecting the generalizability of our findings. One of the study’s limitations is the achieved study power for some parameters (SOD, CAT, MDA) being less than 80%; however, despite this, the majority of results had achieved study power greater than 80%, demonstrating robust statistical reliability for these parameters. The lower power for specific comparisons underscores the necessity for future studies with larger sample sizes to further validate and corroborate our findings. Yet, a significant strength of our study lies in our comprehensive sampling strategy, which includes gingival tissues from patients with amlodipine-induced gingival enlargement, those without this side effect but on amlodipine therapy, and individuals with other causes of gingival enlargement. This approach enriches our molecular insights into the condition. To further enhance the understanding of amlodipine-induced gingival enlargement, future studies should incorporate broader clinical parameters, longitudinal assessments, and functional assays. These additions would provide a more comprehensive view of the underlying mechanisms and help address the limitations identified in our study.

## Figures and Tables

**Table 1:** Sociodemographic characteristics of patients with and without gingival enlargement.

	Total N (%)	Without Gingival Enlargement N (%)	With Gingival Enlargement N (%)	*p*
**Gender**				
Male	11 (34.4)	6 (37.5)	5 (31.2)	1.000
Female	21 (65.6)	10 (62.5)	11 (68.8)	
**Education**				
Elementary	3 (9.4)	0 (0.0)	3 (18.8)	0.130
High School	19 (59.4)	11 (68.8)	8 (50.0)	
College	8 (25.0)	5 (31.2)	3 (18.8)	
Master’s/PhD	2 (6.3)	0 (0.0)	2 (12.5)	
**Employment**				
Yes	18 (56.3)	4 (25.0)	4 (25.0)	0.724
No	8 (25.0)	10 (62.5)	8 (50.0)	
Retired	6 (18.8)	2 (12.5)	4 (25.0)	
**Marital Status**				
Single	3 (9.4)	1 (6.2)	2 (12.5)	0.559
In Relationship	1 (3.1)	1 (6.2)	0 (0.0)	
Married	18 (56.3)	10 (62.5)	8 (50.0)	
Divorced	5 (15.6)	3 (18.8)	2 (12.5)	
Widowed	5 (15.6)	1 (6.2)	4 (25.0)	
**Alcohol Consumption**				
No	23 (71.9)	11 (68.8)	12 (75.0)	1.000
Moderate	9 (28.1)	5 (31.2)	4 (25.0)	
**Physical Activity**				
None	8 (25.0)	2 (12.5)	6 (37.5)	0.352
Occasional	11 (34.4)	6 (37.5)	5 (31.2)	
Regular	13 (40.6)	8 (50.0)	5 (31.2)	
**Comorbidities**				
No	24 (75.0)	11 (68.8)	13 (81.2)	0.685
Yes	8 (25.0)	5 (31.2)	3 (18.8)	
**Additional Therapy**				
No	5 (27.8)	2 (22.2)	3 (33.3)	1.000
Yes	13 (72.2)	7 (77.8)	6 (66.7)	

**Table 2:** Medication, duration, and blood pressure.

	Total N (%)	Without Gingival Enlargement N (%)	With Gingival Enlargement N (%)	*p* **
**Medication Usage**				
No Medication	16 (50.0)	8 (50.0)	8 (50.0)	0.560
Amlodipine	4 (12.5)	3 (18.8)	1 (6.2)	
Combination	12 (37.5)	5 (31.2)	7 (43.8)	
**Dosage**				
No Medication	16 (50.0)	8 (50.0)	8 (50.0)	1.000
5 mg	14 (87.5)	7 (43.8)	7 (43.8)	
10 mg	2 (12.5)	1 (6.2)	1 (6.2)	
	**Total** Mean ± SD	**Without Gingival Enlargement** Mean ± SD	**With Gingival Enlargement** Mean ± SD	*p* ***
Duration of Medication Use (months)	36.44 ± 24.96	14.69 ± 21.75	21.75 ± 29.798	0.440
Disease Duration (years)	12.06 ± 7.69	4.75 ± 7.31	7.31 ± 9.93	0.382
Systolic Blood Pressure *	134.00 ± 10.59	128.38 ± 7.25	139.63 ± 10.743	**0.028**
Diastolic Blood Pressure	85.81 ± 13.51	81.88 ± 7.53	89.75 ± 17.29	0.257

* <0.05; ** Fisher’s exact test; *** independent sample *t*-test.

**Table 3:** Clinical characteristics and indices of patients.

	Without Gingival Enlargement Median (IQR)	With Gingival Enlargement Median (IQR)	*p* **
Age	54.5 (26.75)	50.5 (29.75)	0.956
Charlson Comorbidity Index (CCI)	1.0 (2.0)	0.5 (2.0)	0.724
CCI Percentage	96.0 (8.0)	97.0 (8.0)	0.724
Oral Health Impact Profile—OHIP-14 *	4.0 (7.0)	13.0 (17.0)	**0.002**
WB-HRQoL ^1^	83.0 (12.0)	73.5 (17.0)	0.149
PI	0.92 (1.34)	0.91 (0.69)	0.926
Papillae Bleeding Index *	0.36 (0.92)	0.87 (1.33)	**0.043**
Clinical Attachment Level	1.16 (2.95)	1.1 (1.46)	0.669

* <0.05; ** Mann–Whitney U test; ^1^ Questionnaire for measuring health-related quality of life (HRQoL) in adults residing in western Balkan states.

**Table 4:** Oral health behaviors and symptoms in patients.

	Total N (%)	Without Gingival Enlargement N (%)	With Gingival Enlargement N (%)	*p* **
**Mouth Breathing**				
No	20 (62.5)	12 (75.0)	8 (50.0)	0.273
Yes	12 (37.5)	4 (25.0)	8 (50.0)	
**Bruxism**				
Ne	23 (71.9)	13 (81.2)	10 (62.5)	0.432
Da	9 (28.1)	3 (18.8)	6 (37.5)	
**Parafunctional Habits**				
Ne	26 (81.2)	13 (81.2)	13 (81.2)	1.000
Da	6 (18.8)	3 (18.8)	3 (18.8)	
**Gingival Bleeding**				
No	21 (65.6)	11 (68.8)	10 (65.2)	0.754
Mild	7 (21.9)	4 (25.0)	3 (18.8)	
Moderate	4 (12.5)	1 (6.2)	3 (18.8)	
Masticatory Difficulties *				
No	24 (75.0)	16 (100.0)	8 (50.0)	**0.002**
Yes	8 (25.0)	0 (0.0)	8 (50.0)	
**Speech Difficulties**				
No	29 (90.6)	16 (100.0)	13 (81.2)	0.226
Yes	3 (9.4)	0 (0.0)	3 (18.8)	
**Denture-Wearing Difficulties**				
No	2 (6.2)	1 (6.2)	1 (6.2)	0.792
Yes	4 (12.5)	1 (6.2)	3 (18.8)	
Not Applicable	26 (81.3)	14 (87.5)	12 (75.0)	
**Taste Disorders**				
No	30 (93.8)	16 (100.0)	14 (87.5)	0.484
Yes	2 (6.2)	0 (0.0)	2 (12.5)	
**Halitosis**				
No	21 (65.6)	11 (68.8)	10 (62.5)	1.000
Yes	11 (34.4)	5 (31.2)	6 (37.5)	
**Brushing Twice Daily**				
No	5 (15.6)	3 (18.8)	2 (12.5)	1.000
Yes	27 (84.4)	13 (81.2)	14 (87.5)	
**Mouthwash Usage**				
No	18 (56.2)	10 (62.5)	8 (50.0)	0.722
Yes	14 (43.8)	6 (37.5)	8 (50.0)	
**Dental Floss or Interdental Brushes Usage**				
No	21 (65.6)	11 (68.9)	10 (62.5)	1.000
Yes	11 (34.4)	5 (31.2)	6 (37.5)	
**Regular Check-Ups**				
No	13 (40.6)	7 (43.8)	6 (37.5)	1.000
Yes	19 (59.4)	9 (56.2)	10 (62.5)	

* < 0.05; ** Fisher’s exact test.

**Table 5:** List of primers used in RT-PCR.

Primer Name	Sequence (5′-3′)
Human β-actin	F: AGCACAGAGCCTCGCCTT R: CATCATCCATGGTGAGCTGG
Human IL-6	F: ACTCACCTCTTCAGAACGAATTG R: CCATCTTTGGAAGGTTCAGGTTG
Human TNF	F: CCTCTCTCTAATCAGCCCTCTG R: GAGGACCTGGGAGTAGATGAG
Human VEGF-C	F: ACCTGCCCCACCAATTACA R: GCCTCTTGTAAAGACTGGTT
Human VEGF-D	F: CCTGAAGAAGATCGCTGTTC R: GAGAGCTGGTTCCTGGAGAT
Human TGF β 1	F: CCCAGCATCTGCAAAGCTC R: GTCAATGTACAGCTGCCGCA
Human CTGF	F: GGCTTACCGACTGGAAGAC R: AGGAGGCGTTGTCATTGG
Human IL-33	F: AATCAGGTGACGGTGTTG R: ACACTCCAGGATCAGTCTTG
Human ST2	F: ATGTTCTGGATTGAGGCCAC R: GACTACATCTTCTCCAGGTAGCAT
Human FGF 2	F: CTGGCTATGAAGGAAGATGGA R: TGCCCAGTTCGTTTCAGTG
Human KGF (FGF 7)	F: TTG-TGG-CAA-TCA-AAG-GGG-TG R: CCT-CCG-TTG-TGT-GTC-CAT-TTA-GC
Human EGF	F: CTTGTCATGCTGCTCCTCCTG R: TGCGACTCCTCACATCTCTGC
Human GAPDH	F: GGAAGGTGAAGGTCGGAGTCA R: GTCATTGATGGCAACAATATCCACT

F—forward; R—reverse.

## Data Availability

The data presented in this study are available upon request.
